# The genetics of race differentiation—should it be studied?

**DOI:** 10.1093/nsr/nwad068

**Published:** 2023-03-17

**Authors:** Chung-I Wu

**Affiliations:** Chung-I Wu School of Life Sciences, Sun Yat-sen University, China Associate Editor-in-Chief for Life Sciences at NSR

In this issue, *NSR* publishes an article entitled ‘Expression profiles of east-west highly differentiated genes in Uyghur genomes’ by Ning *et al.* (2023) [1]. Briefly, it is about the regulation of gene expression in Uyghur genomes, which are admixed between Asian and European genomes. In evolutionary terms, this is a case of post-isolation secondary contact over a time span of thousands of years. In most other cases, such as African Americans, the admixture has a time span of <300 years. Furthermore, the genetic contributions from the two parental races are closer to the 1 : 1 ratio than other admixed populations [2–4].

The paper had been reviewed and accepted by a peer journal (Journal A) but, curiously, was declined publication right before the set day of publication (11 January 2023). There was no clear explanation for this unusual action except a vague reference to a task force formed by the American Society of Human Genetics (ASHG) to address the injustice in human genetic research (https://www.ashg.org/publications-news/newsletter/20211215-webinar-confronting-the-past/). It is certain that Journal A was fully aware of the task force and the relevant issues before clearing the study for publication. Hence, the only scientifically legitimate reason for the last-minute reversal, as far as we can fathom, is the larger scientific context that might have eluded the reviews.

This larger context is the ethics of studying race genetics. Races are roughly equivalent to subspecies [5], a taxonomic rank below species but above populations. While the concept of species is controversial in evolutionary biology (see the special *NSR* issue [6]), the controversy over races is more so, to the point of inducing collective muteness on biological races. Races are not stringently delineated for the same reason that no taxonomic rank can be stringently delineated. Even species, often deemed the only rank that has biologically definable characteristics, is no exception (see the so-called ‘species problem’ [6]). Loosely speaking, biological races are geographical populations that have diverged in some phenotypes. If such phenotypes are (or are inferred to be) adaptive and genetically determined, these populations can be regarded as geographical races.

In humans, populations in Africa, Asia and Europe thus qualify as three major human races. Outside of humans, a well-characterized case of racial genetics is the so-called Z race of *Drosophila melanogaster* that is found in southern-central Africa, centered around Zimbabwe [7,8]. The cosmopolitan populations are referred to as the M race and the salient phenotypic difference is that Z females are strongly unreceptive to M males. Genetic and molecular analyses of the two races [9,10] have led to the conclusion that there is extensive genetic divergence, even between racial groups that are morphologically indistinguishable and reproductively fully compatible *post mating*.

When it comes to humans, race genetics is no longer merely scientific questions. The issues are multidimensional, encompassing all aspects of human society: political, religious, cultural and historical. The very first question is simply, ‘Should we study the genetics of human racial differentiation?’ The reservation is expressed by BioScience, Oxford Academic, stating that, ‘Race, as generally accepted by scientists, is not a biological reality but rather reflects the cultural and social underpinning originally used to justify slavery’. The basis of this view may be the caution that science might help to justify racism in human societies. However, in human history, racism was socially acceptable in many societies precisely because of the lack of scientific understanding of the biology of races, as documented by S.J. Gould in his book ‘The Mismeasurement of Man’. The opposite may be true: because of the lack of scientific understanding, every brand of racism can rise without being constrained by that understanding. It is entirely possible that the less scientific understanding we have of the genetics of racial differences, the more biased we are.

There may even be an unstated concern that scientific findings may indeed justify racism. The attitude is itself racist. If one has studied species and race differences, one would have a very different perspective. The genetics is likely to reflect what we already know—that differences in physical attributes such as skin color have a genetic basis. These genetic differences have nothing to do with other traits such as aggression, intelligence, social behaviors, diligence and loyalty. In fact, the genetic bases of such behavioral traits are extremely difficult to pinpoint even in simpler organisms like Drosophila [9,10].

In reality, we have been studying racial genetics in humans with no accompanying controversy. At present, we have tens of thousands of human genomic sequences from a large number of ethnicities [11]. Furthermore, there are many admixed populations among the three major races. People of mixed races have flocked to have their genetic variants determined [12]. In other words, there is no problem in data collection when people consent to have the data collected by themselves or by others. Therefore, it is incorrect to suggest that data collection on human racial differences must be, in principle, ethically questionable. In the specific study of Ning *et al.*, the consent agreements have been meticulously documented and presented with the paper, as microscopic scrutinization by journals and the media was fully anticipated.

In this light, the objections to the Ning *et al.* study likely lie in their analyses and conclusions. This is the main reason why the analyses and conclusions deserve to be read, appreciated and criticized by the academic community. Finally, we address the controversy surrounding Ning *et al.*’s study via a strictly intellectual route. In the event that the objections are not based on scientific and intellectual grounds, there would be an even greater need for a scientific journal to defend science by publishing this study.
